# 

**Published:** 1889-11

**Authors:** 


### CONTENTS—NOVEMBER, 1889—VOL. 5, NO. 5.

ORIGINAL COMMUNICATIONS—

Compound Presentation. R. H. L. Bibb, M. D . . . 163

Report of a Case of Puerperal Eclampsia, with Notes. B. F. Kingsley, M. D . . . . . . . . . . . . . . . 166

Placenta Prævia. P. K. Wortham, M. D. . . . . . . . 171

Preventive Treatment of Urinary Calculi. B. F. Church, M. D . . . . . . . . . . . . . . . . . . . 177

CORRESPONDENCE—

Trachoma. E. P. Edwards, M. D. . . . . . . . . . 184

SOCIETY NOTES—

West Texas District Medical Association, Second Quarterly Meeting; Minutes, etc . . . . . . . . . . . . 185

EDITORIAL—

Medical Department of the University of Texas: Present Status . . . . . . . . . . . . . . . . . . . . . 189

Dr. Merryman Drops In . . . . . . . . . . . . . . 191

MEDICAL NEWS AND MISCELLANY . . . . . . . . . . . 194

BOOK NOTICES . . . . . . . . . . . . . . . . . . . . 197

PUBLISHER'S NOTES . . . . . . . . . . . . . . . . . 198

